# A Fluorescent Probe for the Specific Staining of Cysteine Containing Proteins and Thioredoxin Reductase in SDS-PAGE

**DOI:** 10.3390/bios11050132

**Published:** 2021-04-23

**Authors:** Yuning Liu, Yanan Yu, Qingshi Meng, Xueting Jia, Jiawei Zhu, Chaohua Tang, Qingyu Zhao, Xiaohui Feng, Junmin Zhang

**Affiliations:** 1 State Key Laboratory of Animal Nutrition, Institute of Animal Science, Chinese Academy of Agricultural Sciences, Beijing 100193, China; liuyuning001@126.com (Y.L.); yuyanan@caas.cn (Y.Y.); mengqingshi@caas.cn (Q.M.); jiaxueting2018@163.com (X.J.); zhujiaweisemail@163.com (J.Z.); tangchaohua@caas.cn (C.T.); zhaoqingyu@sina.com (Q.Z.); 2 Scientific Observing and Experiment Station of Animal Genetic Resources and Nutrition in North China of Ministry of Agriculture and Rural Affairs, Institute of Animal Science, Chinese Academy of Agricultural Sciences, Beijing 100193, China

**Keywords:** fluorescent probe, thiol, thioredoxin reductase, SDS-PAGE, labeling, low pH, iodoacetamide, naphthalimide

## Abstract

A naphthalimide-based fluorescent probe, Nap-I, with iodoacetamide as the alkylating group, has been synthesized, and its specific fluorescent staining of proteins containing cysteine (Cys) and selenocysteine (Sec) residues in sodium dodecyl sulfate polyacrylamide gel electrophoresis (SDS-PAGE) has been evaluated. This molecule shows good fluorescence properties in the labeling of protein Cys/Sec residues, while reducing steric hindrance and minimizing changes in the water solubility of proteins. Reaction parameters, such as labeling time and pH, have been investigated, and the optimal labeling conditions for Cys-containing proteins have been determined. Thioredoxin reductase (TXNRD) is best stained at low pH. The probe Nap-I has been successfully used for the quantification of serum proteins and hemoglobin in Tan sheep serum, and TXNRD in Tan sheep liver and muscle has been labeled at low pH. Based on the probe Nap-I, we have also distinguished TXNRD1 and TXNRD2 by SDS-PAGE. The results showed that, compared with the normal microenvironment in which the protein resides, the lower the pH value, the greater the TXNRD activity.

## 1. Introduction

Sulfur and selenium are elements of the same group, and their chemical properties have many similarities. At the same time, sulfur and selenium are also indispensable elements in living organisms. As an important part of amino acids and antioxidant substances in the body, many biomolecules incorporate thiol and selenol moieties, including selenoproteins and some small molecules, such as glutathione (GSH), cysteine (Cys), selenocysteine (Sec), hydrogen selenide (H_2_Se), and so on [1]. Thiols and selenols have strong reducing properties and show very similar biological functions. Both Se in selenols and S in thiols can adopt multiple oxidation forms from −2 to +4, endowing them with versatile reactivity [2]. Thus, selenol and thiols with redox activity are important substances that maintain the stability of the redox state of the microenvironment and buffer important redox changes to regulate cell function and body balance. They provide disease diagnosis and treatment options by participating in some physiological processes and redox reactions. Cells maintain their redox balance based on a well-developed regulatory system. The sub-health state of cells often leads to an increase in reactive oxygen species (ROS) and a change in pH [3]. Selenols with relatively low p*K*_a_ values are more likely to be deprotonated, enabling the reduction of some ROS, thereby serving as anti-oxidants [4,5]. Although the abundance of selenols in organisms is much lower than that of thiols, their extremely strong reducing properties make them the first line of defense in protecting cells from oxidation. Inhibition of thioredoxin reductase (TXNRD, TrxR) can seriously affect the function of the thioredoxin system in cells and cause cell death. Therefore, targeting the TXNRD in cancer cells is a very effective cancer treatment measure [6,7,8]. Selenols and thiols with abnormal levels can lead to poor health, weakened immunity, and various diseases, such as muscle diseases, diabetes, cardiovascular diseases, and neurodegenerative diseases [9]. Since selenols are more easily oxidized than thiols, the monitoring and detection of selenols is more challenging. At the same time, selenols are more active at low pH, which provides a good means of detecting them in tissue samples in vitro [10,11].

Most of the selenol-specific fluorescent probes are adapted versions of thiol-specific probes, such as 2,4-dinitrophenyl [12,13,14], disulfide bond compounds [15,16,17,18], gold-sulfur bonds [19,20], Michael acceptors [21,22,23], benzoselenodiazole [24,25,26,27], and so on [9,28,29,30,31,32]. In fact, the first probe used to detect selenols was adapted from a thiol probe at low pH [11,33]. The p*K*_a_ of selenol is generally low, for Sec is 5.2, but for Cys is 8.5. Since the deprotonation ability of thiols is weakened at low pH, and the deprotonation ability of selenols is enhanced, fluorescent probes with the same detection group may respond better to selenols in a shorter time or at lower pH values. Maleimide [34,35], methyl sulfate [36], and alkyl halides [37] are all good thiol alkylating reagents. As a common thiol alkylation reagent, iodoacetamide (IAA) has become the first choice for labeling thiol-containing proteins and improving the detection signal of trace thiols [38,39]. Indeed, new thiol-labeling fluorescent probes with iodoacetamide as a functional group are constantly being developed. The rapid development of “click chemistry” has also expedited the use of iodoacetamide fluorescent probes [10]. The alkylation efficiency of alkyl halides for thiols decreases at low pH, but the alkylation efficiency for selenols increases. In fact, by using iodoacetamide fluorescent probes to alkylate selenol groups in selenoproteins at low pH, the detection of more selenoproteins can be achieved [10,40,41].

Fluorescent labeling of proteins is a very important method in biochemical detection. In sodium dodecyl sulfate polyacrylamide gel electrophoresis (SDS-PAGE), protein samples are covalently or non-covalently labeled with fluorescent dyes, which can then be used to track and quantify proteins in biological processes. Compared with staining after electrophoresis, prestaining protein samples with covalent labels before electrophoresis will shorten the staining process. Dye-labeled protein typically have a good limit of detection (LOD) and signal-to-noise ratio, and such labeling can be used as a pre-staining method for protein analysis in gels [35,37]. Therefore, our aim was to systematically explore the characteristics of iodoacetamide fluorescent probes suitable for labeling thiols or selenols in different environments, and to provide a convenient and highly selective analytical method.

In this work, we have constructed a fluorescent probe for the detection of proteins containing thiol or selenol moieties. We appended iodoacetamide to naphthimide, thereby constructing a fluorescent probe, Nap-I, for labeling Cys/Sec residues in proteins. We have thoroughly studied the protein-specific staining of Cys residues with Nap-I, and have applied this labeling strategy to the determination of serum albumin and hemoglobin in the serum of Tan sheep. At the same time, Nap-I has been used to specifically stain and distinguish different TXNRDs at low pH, and the TXNRDs in the liver and longissimus thoracis muscles of Se-enriched Tan sheep have been specifically stained.

## 2. Experimental Section

### 2.1. Materials and Instrumentation

All chemical reagents and solvents were purchased from commercial sources (Sigma-Aldrich and Macklin Inc) and were used without further purification. ^1^H and ^13^C NMR spectra were recorded on a Bruker Avance II 300 MHz spectrometer, and chemical shifts are expressed in parts per million (ppm, with respect to DMSO and Me_4_Si as internal standards). Mass spectrum was performed on a Sciex triple TOF 6600 set-up. Absorption spectra were recorded on a Metash UV-8000A ultraviolet-visible spectrophotometer. Fluorescence spectra and quantum yields were measured at room temperature on an FS5 spectrofluorimeter (Edinburgh Instruments). Samples for absorption and fluorescence measurements were placed in a 1 cm × 1 cm quartz cuvette (1 mL volume). Gel fluorescence imaging was performed with a Gel Doc XR+ documentation system (Bio-Rad). Images were analyzed with Quantity One gel image analysis software (Bio-Rad). The enzyme activity of TXNRD in Tan sheep liver and muscle was measured by a commercial kit method [oxidized thioredoxin reductase (TrxR) test kit from Nanjing Jiancheng Bioengineering Institute].

### 2.2. Synthesis of Nap-I

**Scheme 1:**
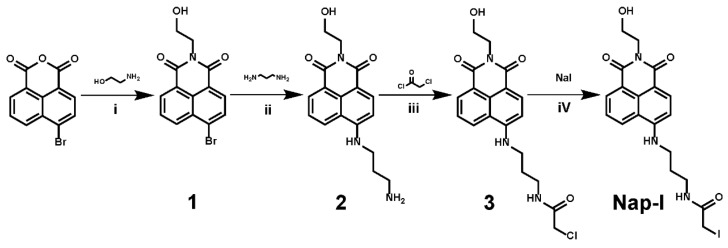
Steps in the synthesis of probe Nap-I: (i) 1,4-dioxane, reflux, 4 h; (ii) 2-methoxyethanol, reflux, 3 h; (iii) CH_2_Cl_2_, triethylamine, room temperature, overnight; (iv) acetone, reflux for 24 h in the dark.

Nap-I was synthesized following the procedure shown in Scheme 1. A mixture of compound 3 (0.389 g, 1 mM) and sodium iodide (1.5 g, 10 mM) in dry acetone (20 mL) was stirred in a flask and heated under reflux for 24 h in the dark under N_2_ protection. After natural cooling, the mixture was filtered and the filtrate was collected. The solvent was evaporated under reduced pressure and the residue was dried and separated by chromatography on a silica gel column (V_DCM_/V_MeOH_ = 10:1) to afford Nap-I (0.45 g, yield: 93%). ^1^H NMR (300 MHz, DMSO-*d*_6_): δ 8.64 (d, *J* = 8.4 Hz, 1H), 8.37 (d, *J* = 7.4 Hz, 1H), 8.20 (d, *J* = 8.5 Hz, 1H), 7.68 (d, *J* = 5.4 Hz, 1H), 7.67–7.59 (m, 1H), 6.74 (d, *J* = 8.6 Hz, 1H), 4.85–4.73 (m, 1H), 4.09 (d, *J* = 6.7 Hz, 1H), 3.63 (s, 1H), 3.57 (d, *J* = 7.0 Hz, 2H), 3.38 (d, *J* = 6.3 Hz, 1H), 3.19 (q, *J* = 6.5 Hz, 1H), 1.83 ppm (p, *J* = 7.0 Hz, 1H); ^13^C NMR (75 MHz, DMSO-*d*_6_): δ 168.22, 164.00, 163.15, 150.51, 134.26, 130.70, 129.45, 128.54, 124.35, 121.88, 120.12, 107.75, 103.89, 58.12, 41.45, 40.44, 40.33, 40.05, 39.77, 39.49, 39.21, 38.93, 38.65, 37.07, 27.75, 1.01 ppm; HRMS (ESI): *m*/*z* calcd for C_19_H_20_IN_3_O_4_ ([*M* + H]^+^): 482.0547; found: 482.0549.

### 2.3. Fluorescence Response Measurements

Fluorescence response of the probe Nap-I to GSH were made in Tris-HCl buffer (100 mM) with 1% DMSO (V_Water_/V_DMSO_ = 99:1). By excitation at 449 nm, fluorescence spectra were recorded in the emission wavelength range of 475 to 700 nm. The appropriate amount of probe was pre-dissolved in DMSO to prepare a 1.0 mM Nap-I stock solution. Calibration measurements were performed by mixing different concentrations of the fluorescent probe Nap-I and GSH in 100 mM Tris-HCl buffer. Specifically, 50.0 μL of a 1 M stock solution of GSH, 1900.0 μL of Tris-HCl buffer solution, and 50.0 μL aliquots of probe solutions of different concentrations were combined to obtain each test solution. For interference experiments, solutions of the various species to be tested (NaCl, KCl, CaCl_2_, MgCl_2_, CuSO_4_, FeCl_3_, ZnCl_2_, HCl, HNO_3_, H_2_SO_4_, vitamin C, H_2_O_2_, HClO, and NaOH) were prepared in doubly-distilled water to give final concentrations of 100 μM.

### 2.4. Determination of Quantum Yield

A solution of Nap-I in doubly-distilled water with 1% DMSO (V_Water_/V_DMSO_ = 99:1) was prepared, and the absorbance at 553 nm was 0.15. An FS5 spectrofluorimeter equipped with an SC-30 Integrating Sphere was used to characterize the absorption and photoluminescence characteristics.

### 2.5. Feeding of Se-Enriched Tan Sheep

Healthy male Tan sheep (*n* = 64) with an average body weight (BW) of 32 ± 1.95 kg were randomly assigned to one of four groups (*n* = 16/group). Based on a basal diet containing 0.16 mg Se/kg and selenium yeast (SeY, 3300 mg Se/kg) as a selenium supplement, the four dietary treatment groups were as follows: (1) basic diet + SeY, 0.25 mg Se/kg, (2) basic diet + SeY, 0.50 mg Se/kg, (3) basic diet + SeY, 1.0 mg Se/kg, and (4) basic diet + SeY, 2.0 mg Se/kg. The basal diet was formulated to meet the daily nutritional requirements for fattening sheep with a weight of 30 kg as described in the NY/T 816-2004 feeding standard of meat-producing sheep and goats, and met the recommended levels of the National Research Council (1985). The amount of supplemented Se in the present trial met the Se requirement of 0.18–0.31 mg/kg (maximum tolerable concentration of 2.0 mg/kg) of the NY/T 816-2004 standard for meat-producing sheep at a BW of 20–50 kg. The Se levels of the basal diet and SeY were determined by inductively coupled plasma mass spectrometry in accordance with the Chinese national standard (GB 5009.268-2016). All sheep were provided with ad libitum access to food and water over the pre-trial period of 10 days and the experimental period of 60 days.

### 2.6. Detection of Proteins

All stained gels were imaged with the Gel Doc XR+ documentation system (Bio-Rad). The images were analyzed with Quantity One gel image analysis software (Bio-Rad). The trace quantity, which represents the intensity (int) of each pixel multiplied by the band area (mm^2^), was plotted versus the amount of protein.

### 2.7. Alkylation of Sec/Cys with Nap-I

Sec, Cys (1 mM each), and a mixture of the two (1 mM each) were dissolved in 1 mL of buffer containing 0.1 m TCEP and 100 mM Tris-HCl (pH 7.4), and reduced for 30 min. The mixture was then diluted with 100 mM Tris-HCl buffer (pH 3.0–9.0), Nap-I (1 μL) was added to give a final concentration of 0.1 mM, and the mixture was vortexed and briefly centrifuged. The system was incubated in the dark at room temperature for 60 min, and then stored prior to LC-MS/MS analysis. The MS signal ratio between the alkylated form and the total intensity was used to calculate the alkylation efficiency at each pH, and in the case of the mixture to calculate the influence of the competition of Sec and Cys in terms of alkylation efficiency at each pH.

## 3. Results and Discussion

### 3.1. Design and Synthesis of Fluorescent Probe Nap-I

As part of our studies on iodoacetamide fluorescent probes for labeling Cys/Sec residues in proteins, we designed the new probe, Nap-I, as shown in Scheme 1. Compound 1 and Compound 2 were synthesized according to reported methods with minor changes [42,43]. Specifically, a naphthalimide moiety serves as the fluorophore and iodoacetamide is used as an alkylating group of Cys/Sec residues. In order to enable the fluorescent probe Nap-I to better alkylate Cys/Sec residues in proteins, we minimized the quenching effect of iodine on the fluorophore, and used 1,3-propanediamine as a linker to reduce the possible steric hindrance caused by the alkylation of Cys/Sec residues with the probe. At the same time, the appending of a hydroxyl group reduces the effect of the probe on the solubility of proteins in water. Other promising strategies for the fluorescent labeling of thiols have recently been described (Table S1) [34,35,36,37,38,39,44,45,46].

The new probe Nap-I was synthesized according to the route shown in Scheme 1. Commercially available 4-bromo-1,8-naphthalic anhydride was first treated with ethanolamine to produce compound 1, which was then reacted with 1,2-ethylenediamine to produce compound 2. Compound 2 was then reacted with chloroacetyl chloride to produce compound 3, the exchange of which with sodium iodide generated probe Nap-I. The product was characterized by ^1^H/^13^C NMR spectroscopy and ESI-MS. Figures S1–S6 show the relevant spectra at each stage of the synthesis.

### 3.2. Spectrophotometric Measurements of Fluorescent Probe Nap-I

**Figure 1:**
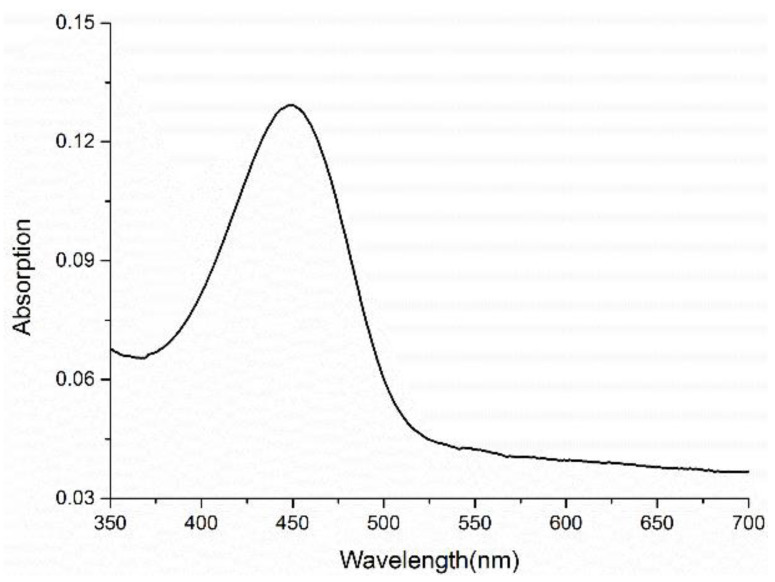
Absorption spectra of 100 μm Nap-I in 100 mM Tris-HCl buffer with 1% DMSO as co-solvent (V_Water_/V_DMSO_ = 99:1).

**Figure 2:**
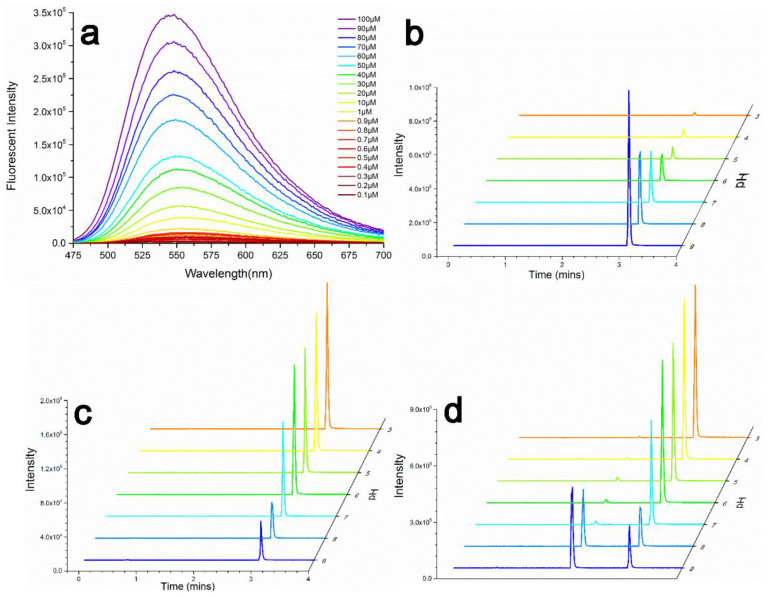
(**a**) fluorescence emission spectra of 10 mM GSH in 100 mM Tris-HCl buffer with 1% DMSO as co-solvent (V_Water_/V_DMSO_ = 99:1) at different Nap-I concentrations in the range 0–100 μM; (**b**) alkylation efficiency of Cys by Nap-I at different pH; (**c**) alkylation efficiency of Sec by Nap-I at different pH; (**d**) competitive alkylation efficiencies of Cys and Sec by Nap-I.

The UV/Vis and fluorescence characteristics of Nap-I were studied in 100 mM Tris-HCl buffer (V_Water_/V_DMSO_ = 99:1). As shown in Figure 1, the fluorescent probe Nap-I has an obvious absorption peak near 449 nm. When different concentrations of Nap-I (0–100 μM) were incubated with 10 mM GSH (thiol donor), the emission wavelength was 553 nm. As the concentration of Nap-I was increased (0–100 μM), its emission intensity at 553 nm gradually increased, implying that alkylation of the thiol moiety on GSH by the probe does not affect its fluorescence emission (Figure 2a).

The quantum yield was studied for 15 μM Nap-I in 100 mM Tris-HCl buffer (V_Water_/V_DMSO_ = 99:1). The probe Nap-I maintains the high quantum yield of the naphthalimide fluorophore, which was calculated to be 67.7% (Figure S7).

Anti-interference ability is a key aspect for measuring new probes. Thus, to study the anti-interference ability of Nap-I, it was incubated with various cations, anions, and ROS, such as NaCl, KCl, CaCl_2_, MgCl_2_, CuSO_4_, FeCl_3_, ZnCl_2_, HCl, HNO_3_, H_2_SO_4_, vitamin C, H_2_O_2_, HClO, and NaOH, each at 10 mM. It can be seen from Figure S8 that when excited at 445 nm, Nap-I gives rise to a significant increase in fluorescence intensity at 553 nm, and that this is largely unaffected by the presence of other analytes. Next, the effect of pH on Nap-I and Nap-GSH was studied (Figure S9). No obvious changes in fluorescence intensity were observed in 100 μM Nap-I and Nap-GSH solutions in the range pH 3.0–9.0. The results show that Nap-I can label thiols over a wide pH range, and that this is not affected by various species.

Photostability is one of the important parameters of tagged fluorescent probes. We exposed Nap-GSH (100 μM, Tris-HCl pH = 7.4) to a 150-W ozone-free xenon lamp for 8 h to test the photostability of the probe after labeling. The probe inherits the stable resonance structure of the naphthalimide fluorophore, and even after being irradiated for eight hours, the residual fluorescence intensity can reach 90% of the initial value (Figure S10).

### 3.3. Alkylation Efficiency of Sec/Cys with Nap-I at Different pH

The results show that Nap-I can label thiols over a wide pH range, and that this is not affected by various species. The alkylation efficiencies of Sec/Cys with Nap-I at different pH (3.0–9.0) were tested. The p*K*_a_ of Cys is 8.5, and its alkylation efficiency gradually increases with increasing pH (Figure 2b). Conversely, the alkylation efficiency of Sec gradually increases with decreasing pH (Figure 2c). ESI-MS of corresponding alkylated derivatives of Nap-I to Cys/Sec are shown in Figures S5 and S6.

When Sec and Cys co-exist, as the pH is decreased, the competitive alkylation efficiency of Nap-I for Sec gradually increases, reaching 99.27% at pH 3.0 (Figure 2d). In a system without Sec, Nap-I can still efficiently alkylate Cys at pH 7.0. However, in the presence of Sec, its competitive alkylation efficiency can exceed 95% at pH < 7.0 (especially at around pH 5.2). Although the alkylation efficiency of Sec decreases at pH 3.0, its competitive alkylation efficiency improves due to a greater decrease in the alkylation efficiency of Cys.

### 3.4. Labeling of Thiol-Containing Proteins by Nap-I

**Figure 3:**
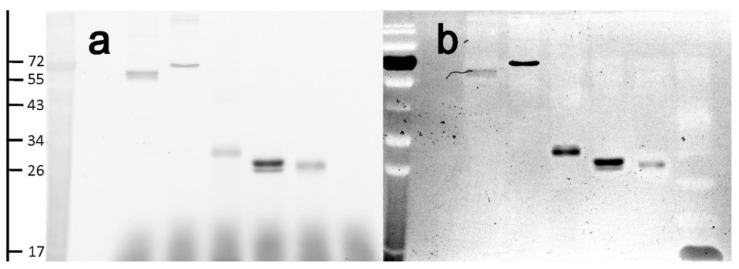
(**a**) Inverted SDS-PAGE fluorescence image of different proteins with Cys residues labeled with Nap-I; (**b**) image of the same gel re-stained with Coomassie R-250. Quantity of protein per band: ca. 1.5 μg.

Specific labeling of Cys residues by Nap-I was examined through the labeling of proteins with different Cys contents. TXNRD from mouse liver (single active Cys, 54.8 kDa TXNRD1 and 56.6 kDa for TXNRD2), bovine serum albumin (BSA) (single active Cys, 66.4 kDa), bovine hemoglobin (single active Cys, 64.5 kDa), bromelain (single active Cys, 33.0 kDa), papain (single active Cys, 23.4 kDa), and equine myoglobin (no active Cys, 17.6 kDa) were each labeled with 0.5 mM Nap-I. The corresponding fluorescent products were analyzed by gel electrophoresis (Figure 3a). The proteins containing active Cys residues all showed better fluorescence intensity, but different proteins may behave slightly differently due to their enzyme activity conditions and domain exposures. TXNRD is a selenoprotein containing both thiol and selenol groups, and it also shows good fluorescence intensity. Hemoglobin in the blood is in equilibrium between dimers and tetramers, becoming a dimer after deoxygenation. 2,3-Diphosphoglyceric acid can bind to deoxyhemoglobin through salt bonds, without the involvement of disulfide bonds. This equilibrium is dependent on temperature, pH, and anions, so its dimer only shows a band at 32 kDa [47,48,49]. As shown in Figure 3b, equine myoglobin, a protein with no Cys residues in its structure and no fluorescence at 449 nm, can be visualized by Coomassie Brilliant Blue R250 staining.

**Figure 4:**
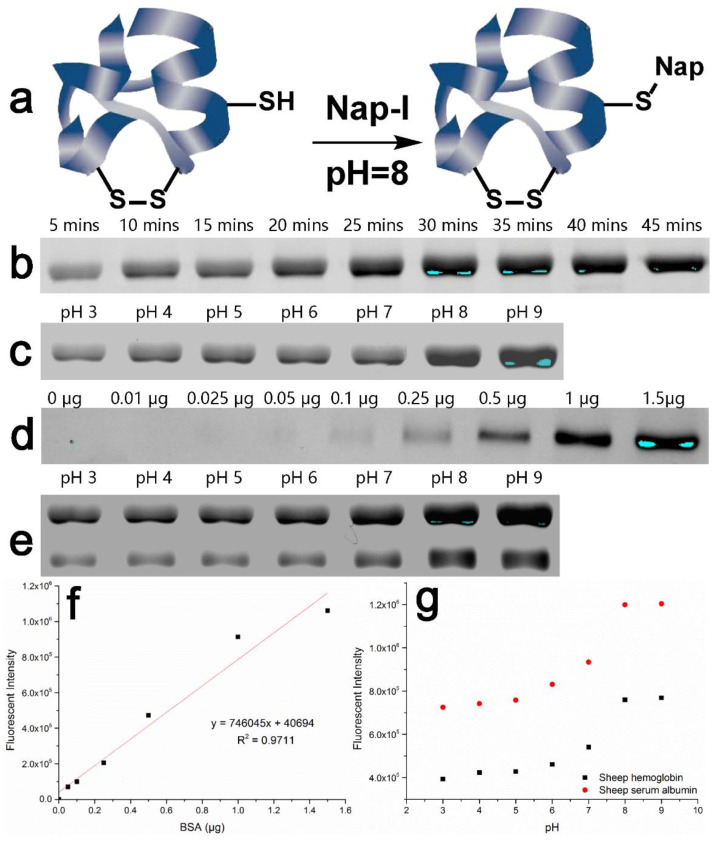
(**a**) Mechanism of the labeling of Cys-containing proteins by Nap-I; (**b**) SDS-PAGE fluorescence image of Nap-I-labeled BSA with different labeling times; (**c**) SDS-PAGE fluorescence image of Nap-I-labeled BSA with different labeling pH; (**d**) SDS-PAGE fluorescence image of Nap-I-labeled BSA in different quantities; (**e**) SDS-PAGE fluorescence image of Nap-I-labeled main proteins with different labeling pH; (**f**) calibration plot of Nap-I fluorescence vs. amount of BSA in the range 0–1.5 μg and linear relationship; (**g**) comparison of Nap-I-labeled sheep serum albumin and sheep hemoglobin at different pH. Quantity of protein per band except Figure 3d: ca. 1.5 μg.

We labeled BSA samples with Nap-I at room temperature for durations of 5–45 min at pH 7.4. The fluorescence intensity of pre-stained BSA increased with extension of the labeling time. The fluorescence of the compound reached its maximum intensity at 30 min, and thereafter remained unchanged when the reaction time was extended for a further 15 min (Figure 4b). According to this result, the reaction time should be controlled at about 30 min to avoid any undesirable non-specific labeling. By reacting equal amounts of BSA with Nap-I in Tris-HCl buffer solutions at different pH in the range 3.0–9.0 for 30 min, it was established that pH 8.0–9.0 is the optimal reaction environment for a reaction time of 30 min (Figure 4c). Moreover, as the amount of BSA was increased from 0 to 1.5 μg, a good linear response was seen (FI = 746,045 × [BSA] μg + 40,694; *R*^2^ = 0.9711), and the limit of detection (LOD) was calculated to be as low as 37.5 nM (2.5 mg/L) (Figure 4d,f).

### 3.5. Determination of the Main Thiol-Containing Proteins in the Serum of Tan Sheep

Serum (1 μL) from the fourth group of Tan sheep was mixed with Nap-I in Tris-HCl buffer of different pH (3.0–9.0). The labeling was found to be incomplete after a reaction time of 30 min. Both Tan sheep serum albumin (69.19 kDa) and hemoglobin (64.29 kDa) bear active thiol groups, and are the main thiol-containing proteins in Tan sheep serum. The optimal labeling time here was about 1 h (Figure 4e,g). The increased time requirement may be due to the enzyme activity conditions of different proteins and the matrix effect in the actual sample. However, if the reaction time was too long, the Tan sheep serum protein was labeled abnormally at low pH, and if it was too short, only the bands at pH 8.0–9.0 appeared (Figures S11 and S12). It was established that pH 8.0 is optimal for detection; the Tan sheep serum albumin content was 38.82 g/L and the hemoglobin content was 24.1 g/L.

### 3.6. Differentiation and Labeling of TXNRD1 and TXNRD2 in Mouse Liver

**Figure 5:**
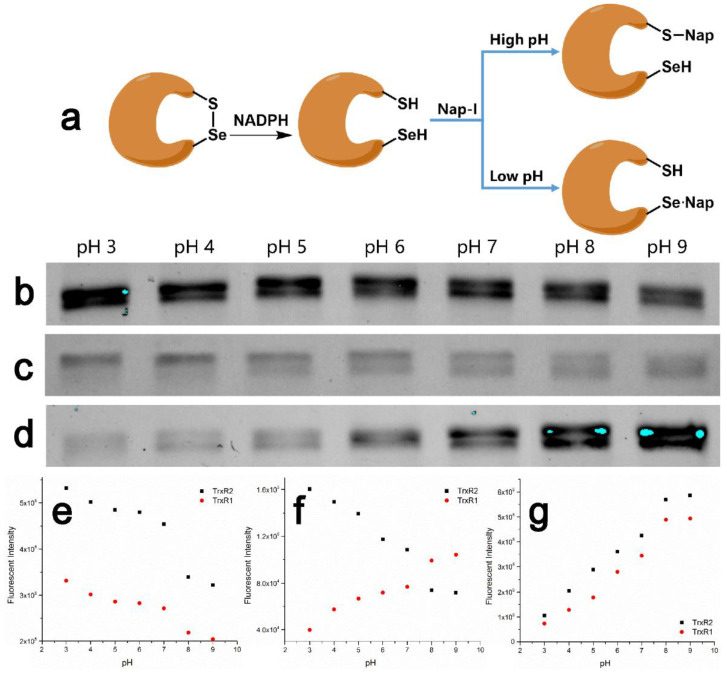
(**a**) Mechanism of the labeling of TXNRD by Nap-I at different pH; (**b**) SDS-PAGE fluorescence image of Nap-I-labeled TXNRD from mouse liver reduced by NADPH at pH 6.5 with different labeling pH; (**c**) SDS-PAGE fluorescence image of Nap-I-labeled TXNRD from mouse liver reduced by NADPH at pH 7.4 with different labeling pH; (**d**) SDS-PAGE fluorescence image of Nap-I-labeled TXNRD from mouse liver reduced by NADPH at pH 8.5 with different labeling pH; (**e**) comparison of Nap-I-labeled TXNRDs from mouse liver reduced by NADPH at pH 6.5 at different pH; (**f**) comparison of Nap-I-labeled TXNRDs from mouse liver reduced by NADPH at pH 7.4 at different pH; (**g**) comparison of Nap-I-labeled TXNRDs from mouse liver reduced by NADPH at pH 8.5 at different pH. Quantity of protein per band: ca. 1 μg.

There are two main bands in commercially available mouse liver thioredoxin reductase, TXNRD1 (54.8 kDa, from cytoplasm) and TXNRD1 (56.6 kDa, from mitochondria). Although there is no effect on the activity measurement of TXNRD, the two main proteins seem to have a negative effect on quantification. Nicotinamide adenine dinucleotide phosphate (NADPH) can be used to reduce TXNRD, but not other selenoproteins. TXNRD is an NADPH-dependent dimeric selenase containing flavin adenine dinucleotide (FAD) domains. It is a member of the pyridine nucleotide-disulfide oxidoreductase family and forms the thioredoxin (Trx) system together with Trx and NADPH [6,7,8,17]. However, due to a difference in microenvironments in cells, the enzyme activity conditions of TXNRD1 and TXNRD2 are slightly different. The pH of the cytoplasm and mitochondria are 7.4 and 8.0, respectively. We specifically reduced TXNRD by NADPH at three different pH values (6.5, 7.4, 8.5), and incubated the reduced TXNRD with Nap-I at different pH values of 3.0–9.0 (Figure 5a–d).

TXNRD was reduced by NADPH at pH 6.5 (Figure 5b,e). The exposure of the domain and the selenium-sulfur bond breakage at low pH make Sec residues easier to deprotonate and then alkylate by Nap-I, whereas Cys residues are difficult to alkylate at low pH. Whereas the fluorescence intensity at high pH was relatively weak, it became higher at low pH, and the band resembled the anti-BSA band. The extent of alkylation of Cys residues by Nap-I decreases at low pH, but the alkylation of Sec is favored. Nap-I mainly alkylates Cys at high pH, but because TXNRD is reduced in a weakly acidic environment, the possibility of Cys alkylation is low. The p*K*_a_ of selenol is about 5.2, and TXNRD is more active in the weakly acidic environment of cancer cells, which is related to deprotonation of the Sec residue of TXNRD at low pH. The high activity of TXNRD at low pH is one of the key factors permitting unlimited expansion of cancer cells [6,7,8].

When TXNRD was reduced at pH 7.4 (Figure 5c,f), although TXNRD1 is at normal cytoplasmic pH and the selenium-sulfur bond was broken, the domain may not have been completely exposed. The groups alkylated by Nap-I are mostly Cys residues, which have almost no fluorescence intensity at low pH, so the band for TXNRD1 appeared similar to that for BSA. It is abnormal for TXNRD2 in the environment under pH 7.4, so the TXNRD2 domain has a certain degree of exposure. This is conducive to the alkylation of Sec residues by Nap-I, but the fluorescence intensity of TXNRD2 at low pH is weaker than that following reduction at pH 6.5. This implies that not all TXNRD2 domains were exposed and not all Sec residues were deprotonated, so the band for TXNRD2 appears similar to the anti-BSA band. Therefore, although both TXNRD1 and TXNRD2 contain selenium-sulfur bonds and are reduced in the same way, they are still sensitive to different cell microenvironments. This is probably one of the reasons for the loss of mitochondrial function in cancer cells and the relatively active mitochondrial TXNRD2 [50]. ROS are mainly produced and consumed in mitochondria [51,52,53]. TrxR2 plays a key role when the Trx system consumes ROS, which is an indispensable part of maintaining mitochondrial function [54]. Insomnia disorders are one of the main manifestations of sub-health. Many cancer patients suffer from insomnia disorders in the early stages of onset. Studies have shown that the expression of TrxR2 is significantly higher in patients with insomnia disorders than in those with normal sleep [55]. Therefore, the increased expression of TrxR may be conducive to the early diagnosis of diseases such as cancer.

When TXNRD was reduced at pH 8.5 (Figure 5d,g), although the selenium-sulfur bond may have been broken, since the pH of the environment was higher than that of the cell microenvironment in which the components reside, the Sec residues were not extensively deprotonated, and thus, were not readily alkylated by Nap-I. At the same time, most of the residues alkylated by Nap-I were Cys residues, so both proteins gave bands resembling those of BSA.

Because the two proteins are in different cell microenvironments, the conditions for activation are slightly different. The environmental pH when TXNRD is reduced by NADPH is one of the factors that may be influenced by selenol after deprotonation.

Compared with the situation at pH 7.4, when TXNRD2 is activated by NADPH at pH 6.5, the fluorescence intensity is greater and the residues are more easily alkylated by Nap-I, this pH being lower than that of the microenvironment of TXNRD2 in mitochondria (pH 8.0). Therefore, the lower the pH, the more active the TXNRD. However, regardless of the reduction of TXNRD at any of the above pH values, an excessively long reaction time (such as 1 h) will result in similar bands for TXNRD1 and TXNRD2 without a gradient (Figure S13). It is helpful to distinguish TXNRD1 and TXNRD2 at pH 7.4. At the same time, the relative quantification of total TXNRD is desirable at a lower pH.

### 3.7. Labeling of TXNRD in Tan Sheep Liver and Muscle

**Figure 6:**
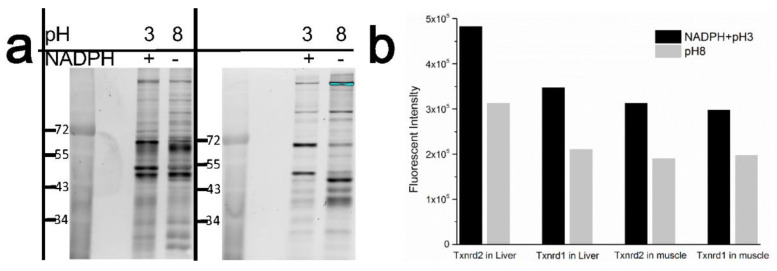
(**a**) SDS-PAGE fluorescence image of Nap-I-labeled TXNRD from Tan sheep liver and muscle reduced by NADPH at pH 6.5 with labeling at pH 3 and 8; (**b**) comparison of Nap-I-labeled TXNRDs from Tan sheep liver and muscle, with or without reduction and labeling at low pH. Quantity of protein per band: ca. 1 μg.

Next, compared with unreduced TXNRD labeled at pH 8, TXNRDs from Tan sheep liver and muscle were reduced with NADPH at pH 6.5 and labeled with Nap-I at pH 3 (Figure 6a,b). It can be seen that both TXNRD1 (66.97 kDa) and TXNRD2 (50.05 kDa) showed higher fluorescence intensity at low pH than at high pH, whereas the other bands had lower fluorescence intensity at low pH than at high pH. The total enzyme activity of TXNRD in Tan sheep liver and muscle were calculated by a commercial kit method as 7.55 u/g and 5.23 u/g, respectively. By comparing the integrated optical densities (IODs), the ratio of the sum of the IODs of the two TXNRD bands in the liver and muscle could be calculated. The ratio of the total TXNRD enzyme activities of the liver and muscle calculated by the commercial kit method was 101.99%, based on Equation (1). The deviation between this method and the kit method was only 1.99%, which shows that the present method has the potential to achieve relative quantification of TXNRD.

(1) $$\mathrm{The}\;\mathrm{ratio}=\frac{\frac{\mathrm{IOD}\;\mathrm{of}\;\mathrm{TXNRD}1\;+\;\mathrm{TXNRD}2\;\mathrm{in}\;\mathrm{liver}\;}{\mathrm{IOD}\;\mathrm{of}\;\mathrm{TXNRD}1\;+\;\mathrm{TXNRD}2\;\mathrm{in}\;\mathrm{muscle}}}{\frac{\mathrm{TXNRD}\;\mathrm{enzyme}\;\mathrm{activity}\;\mathrm{of}\;\mathrm{liver}}{\mathrm{TXNRD}\;\mathrm{enzyme}\;\mathrm{activity}\;\mathrm{of}\;\mathrm{muscle}}}\times 100\%$$

## 4. Conclusions

In conclusion, a novel thiol/selenol-labeling fluorescent probe, Nap-I, has been prepared and evaluated. Probe Nap-I has an excellent signal-to-noise ratio in SDS-PAGE staining applications. By studying the reaction parameters, such as labeling time and pH, the optimal labeling conditions for Cys residues in the protein have been determined, and various proteins containing Cys residues have been labeled. The Sec residues in the protein were labeled under conditions of low pH. The LOD of the Nap-I probe for BSA in SDS-PAGE is 37.5 nM, and it has been successfully used for the quantification of serum protein and hemoglobin in Tan sheep serum. Based on the probe Nap-I, TXNRD in Tan sheep liver and muscle was labeled at low pH, and TXNRD1 and TXNRD2 could be distinguished. Since the thiol moieties in TXNRD still contribute to the fluorescence intensity, it is difficult to quantify TXNRD only by Nap-I absolutely.

## Supplementary Materials

The following are available online at https://www.mdpi.com/article/10.3390/bios11050132/s1, Table S1: Fluorescent probes for thiol labeling, Figure S1. ^1^H and ^13^C NMR spectra of compound 1, Figure S2. ^1^H and ^13^C NMR spectra of compound 2, Figure S3. ^1^H and ^13^C NMR spectra of compound 3, Figure S4. ^1^H and ^13^C NMR spectra and mass spectrum of Nap-I, Figure S5. Mass spectrum of the alkylation product of Nap-I and Cys, Figure S6. Mass spectrum of the alkylation product of Nap-I and Sec, Figure S7. Quantum yield of Nap-I, Figure S8. Interference experiments: fluorescence responses of 100 μm Nap-I in 100 mM Tris-HCl buffer with 1% DMSO as co-solvent (V_Water_/V_DMSO_ = 99:1) in the presence of 10 mM of potentially interfering species, namely NaCl, KCl, CaCl_2_, MgCl_2_, CuSO_4_, FeCl_3_, ZnCl_2_, HCl, HNO_3_, H_2_SO_4_, vitamin C, H_2_O_2_, HClO, and NaOH, Figure S9. pH stabilities of 100 μm Nap-I and 100 μm Nap-GSH, Figure S10. Photostability of 100 μm Nap-I and 100 μm Nap-I, Figure S11. SDS-PAGE fluorescence image of Nap-I-labeled main proteins with different labeling pH (reaction in 30 min), Figure S12. SDS-PAGE fluorescence image of Nap-I-labeled main proteins with different labeling pH (reaction in 1 h), Figure S13. SDS-PAGE fluorescence image of Nap-I-labeled TXNRD from mouse liver reduced by NADPH with different labeling pH (reaction in 1 h).
