# Supplementary material for: A Fluorescent Probe for the Specific Staining of Cysteine Containing Proteins and Thioredoxin Reductase in SDS-PAGE
Source: Biosensors (Basel). 2021 Apr 23;11(5):132. doi: 10.3390/bios11050132 (PMC8146322; doi:10.3390/bios11050132)
Supplement: Supplementary file 1 [file biosensors-11-00132-s001.zip › biosensors-1159189-supplementary.pdf]

biosensors

MDPI

A Fluorescent Probe for the Specific Staining of Cysteine Containing Proteins and Thioredoxin Reductase in SDS-PAGE

Yuning Liu [1,2], Yanan Yu [1,2], Qingshi Meng [1,2], Jiawei Zhu [1,2], Xueting Jia [1,2], Qingyu Zhao [1,2], Chaohua Tang [1,2], Xiaohui Feng [1,2,*] and Junmin Zhang [1,2,*]

1 State Key Laboratory of Animal Nutrition, Institute of Animal Science, Chinese Academy of Agricultural Sciences, Beijing 100193, China; liuyuning001@126.com (Y.L.); yuyanan@caas.cn (Y.Y.); mengqingshi@caas.cn (Q.M.); zhujiaweisemail@163.com (J.Z.); jiaxueting2018@163.com (X.J.); zhaoqingyu@sina.com (Q.Z.); tangchaohua@caas.cn (C.T.)

2 Scientific Observing and Experiment Station of Animal Genetic Resources and Nutrition in North China of Ministry of Agriculture and Rural Affairs, Institute of Animal Science, Chinese Academy of Agricultural Sciences, Beijing 100193, China

* Correspondence: fengxiaohui@caas.cn (X.F.); zhjmxms@sina.com (J.Z.)

Citation: Liu, Y.; Yu, Y.; Meng, Q.; Zhu, J.; Jia, X.; Zhao, Q.; Tang, C.; Feng, X.; Zhang, J. A Fluorescent Probe for the Specific Staining of Cysteine Containing Proteins and Thioredoxin Reductase in SDS-PAGE. *Biosensors* **2021**, *11*, 132. https://doi.org/10.3390/bios11050132

Received: 18 March 2021
Accepted: 21 April 2021
Published: 23 April 2021

Publisher's Note: MDPI stays neutral with regard to jurisdictional claims in published maps and institutional affiliations.

Copyright: © 2021 by the authors. Licensee MDPI, Basel, Switzerland. This article is an open access article distributed under the terms and conditions of the Creative Commons Attribution (CC BY) license (http://creativecommons.org/licenses/by/4.0/).

1. Experimental Part

1.1. Synthesis of Compounds 1–3

Synthesis of compound 1. A mixture of 4-bromo-1,8-naphthalic anhydride (2.77 g, 10 mM) and 2-ethanolamine (0.64 mL, 10.5 mM) in dry 1,4-dioxane (50 mL) was stirred in a flask and heated under reflux for 4 h. After natural cooling, the mixture was filtered, and the filter cake was collected and washed with cold ethanol to obtain a light-yellow solid (2.85 g, yield: 89%), which could be directly used in the next reaction. ^{1}H NMR (300 MHz, DMSO-d_6): δ 8.44 (dd, J = 12.3, 7.9 Hz, 2H), 8.22 (d, J = 7.9 Hz, 1H), 8.11 (d, J = 7.9 Hz, 1H), 7.97–7.85 (m, 1H), 4.82 (t, J = 6.0 Hz, 1H), 4.10 (t, J = 6.5 Hz, 2H), 3.61 ppm (q, J = 6.4 Hz, 2H); ^{13}C NMR (75 MHz, DMSO-d_6): δ 163.03, 162.99, 132.54, 131.55, 131.37, 130.93, 129.79, 129.06, 128.80, 128.32, 122.88, 122.10, 57.84, 42.07, 40.48, 40.21, 39.93, 39.65, 39.37, 39.10, 38.82 ppm; HRMS (ESI): m/z calcd for $C_{14}H_{10}BrNO_3$ ([M+H]$^+$): 319.9900; found: 319.9920.

Synthesis of compound 2. A mixture of compound **1** (1.6 g, 5 mM) and 1,3-propanediamine (8.3 mL, 50 mM) in dry 2-methoxyethanol (50 mL) was stirred in a flask and heated under reflux for 3 h. After natural cooling, the mixture was filtered, and the filter cake was collected and dried under reduced pressure. It was recrystallized from chlorobenzene to obtain an orange solid (1.45 g, yield: 93%), which could be directly used in the next reaction. ^{1}H NMR (300 MHz, DMSO-d_6): δ 8.57 (d, J = 8.4 Hz, 1H), 8.36 (d, J = 7.3 Hz, 1H), 8.20 (d, J = 8.5 Hz, 1H), 7.61 (t, J = 7.9 Hz, 1H), 6.70 (d, J = 8.6 Hz, 1H), 4.09 (t, J = 6.8 Hz, 2H), 3.57 (t, J = 6.8 Hz, 3H), 3.40 (t, J = 6.9 Hz, 4H), 2.71 (t, J = 6.4 Hz, 2H), 1.76 ppm (p, J = 6.7 Hz, 2H); ^{13}C NMR (75 MHz, DMSO-d_6): δ 164.00, 163.13, 150.78, 134.31, 130.63, 130.38, 129.54, 128.47, 124.22, 122.01, 120.17, 107.58, 103.70, 58.13, 41.48, 41.42, 40.49, 40.21, 39.93, 39.74, 39.65, 39.38, 39.10, 38.82, 31.32 ppm; HRMS (ESI): m/z calcd for $C_{17}H_{19}N_3O_3$ ([M+H]$^+$): 314.1408; found: 314.1508.

Synthesis of compound 3. A mixture of compound **2** (1.55 g, 5 mM) and triethylamine (8.3 mL, 50 mM) in dry CH_2Cl_2 (50 mL) was stirred under N_2 in a flask immersed in an ice bath. A solution of chloroacetyl chloride (1.6 mL, 20 mM) in CH_2Cl_2 was then slowly added dropwise. After the addition, the system was allowed to warm to room temperature and the reaction was allowed to proceed overnight. The solvent was then evaporated under reduced pressure and the residue was dried. It was separated by chromatography on a silica gel column (V_{DCM}/V_{MeOH} = 10:1) to afford **3** (1.5 g, yield: 76%). ^{1}H NMR (300 MHz, DMSO-d_6): δ 8.63 (d, J = 7.5 Hz, 1H), 8.38 (d, J = 6.4 Hz, 1H), 8.33 (d, J = 5.7 Hz, 1H), 8.21 (d, J = 8.5 Hz, 1H), 7.68 (d, J = 7.9 Hz, 1H), 7.62 (d, J = 8.4 Hz, 1H), 6.73 (d, J = 8.7 Hz, 1H), 4.79 (t, J = 5.9 Hz, 1H), 4.11 (d, J = 6.8 Hz, 2H), 4.07 (s, 2H), 3.57 (q, J = 6.5 Hz, 2H), 3.38 (d, J = 6.4 Hz, 2H), 3.25 (q, J = 6.5 Hz, 2H), 1.86 ppm (p, J = 6.9 Hz, 2H); ^{13}C NMR (75 MHz,

DMSO-d_6): δ 166.20, 163.97, 163.12, 150.54, 134.21, 130.67, 129.52, 128.49, 124.30, 122.06, 120.22, 107.93, 103.81, 58.12, 42.81, 41.50, 40.57, 40.50, 40.23, 39.95, 39.67, 39.39, 39.11, 38.84, 37.08, 27.83 ppm; HRMS (ESI): *m*/*z* calcd for $C_{19}H_{20}ClN_3O_4$ ([*M*+H]$^+$): 390.1108; found: 390.1149.

1.2. Preparation of Nap-I Solution

Nap-I (4.801 mg) was dissolved in DMSO (1 mL). The labeling reagent stock solution was stored at -20 °C.

1.3. Preparation of Nap-GSH Solution

Aqueous GSH solution (200 µL, 10 mM) and Nap-I solution (200 µL, 1 mM) were combined in a 1.5 mL Eppendorf tube. Tris-HCl buffer (pH 7.4, 250 µL) and acetonitrile (175 µL) were then added, and the mixture was well shaken. The reaction was allowed to proceed for 30 min at 45 °C. After cooling, the volume was adjusted to 2.0 mL with acetonitrile to obtain a 0.1 mM Nap-GSH stock solution. At this time, thin-layer chromatography showed that the reagent had completely reacted, and that only its GSH derivative Nap-GSH was present.

1.4. Collection and Preparation of Biological Samples from Se-Enriched Tan Sheep

Serum was collected from the jugular vein of each sheep after fasting for 12 h at the end of the experiment. It was centrifuged at 4 °C and 3000 rpm for 10 min. Eight sheep of each group of similar weight were selected for slaughter at the end of the experiment and after 12 h of fasting; their longissimus thoracis muscles and livers were collected and immediately frozen in liquid nitrogen and stored at -80 °C until analysis.

Liver samples and longissimus dorsi muscle samples from Tan sheep (0.5 g each) were dissolved in 200 µL RIPA lysis buffer, homogenized, and centrifuged, and then the supernatant was taken. It was diluted in RIPA buffer and then reduced with NADPH for 30 min at 37 °C to obtain the respective reduced thioredoxin reductase (0.5 mg/mL). Tan sheep serum could be used directly.

1.5. Preparation of Protein Samples

BSA, bovine hemoglobin, bromelain, papain, and myoglobin from equine heart (1 mg each) were respectively dissolved in 1 mL of buffer containing 8 M urea and 100 mM Tris (pH 8.5) and then reduced with Tris(2-carboxyethyl)phosphine (TCEP, 1 mL, 0.5 M) for 30 min at 37 °C to obtain various reduced proteins (0.5 mg/mL).

TXNRD from rat liver (5 mg/mL) was reduced with NADPH for 30 min at 37 °C at different pH (6.5, 7.4, and 8.5) to obtain various reduced TXNRD (0.5 mg/mL).

Liver samples and longissimus dorsi muscle samples from Tan sheep (0.5 g each) were dissolved in 200 µL RIPA lysis buffer, homogenized, and centrifuged, and then the supernatant was taken. Tan sheep serum could be used directly after being diluted 25-fold.

1.6. General Fluorescent Labeling Protocol and Gel Electrophoresis

The reduced protein sample (5 µL, 0.5 µg/µL), Tris-HCl buffer of different pH (10 µL, 100 mM), and Nap-I (1 µL, 1 µg/µL) were combined in a 200 µL tube, then vortexed and briefly centrifuged. The system was incubated in the dark at room temperature for 60 min. The loading buffer (4 µL, including DTT) was then added and mixed well. The system was heated for 5 min at 95–100 °C. The samples were then either cooled to room temperature for instant use or frozen for eventual use.

biosensors MDPI

Serial dilutions of the proteins were labeled and loaded onto the gel lanes. Electrophoresis was carried out on self-cast polyacrylamide mini-gels (1 mm thick) using a discontinuous buffer system. The separation gel (pH 8.8) contained 10% polyacrylamide. The stacking gel (pH 6.8) contained 4% polyacrylamide. The running buffer contained 25 mM Tris (pH 8.6), 192 mM glycine, and 0.1% SDS (w/v) in water. All solutions were freshly prepared prior to use. SDS-PAGE was carried out on a vertical polyacrylamide gel system at a current of 15 mA until the protein bands reached the interface of the separating gel. Separation was performed at 115 V for 1.5 h.

1.7. Coomassie Brilliant Blue (CBB) Post-Staining and Destaining Protocol

Coomassie blue R250 (2.5 g) was dissolved in methanol/acetic acid/water (5:1:4, v/v/v; 1000 mL), with stirring as needed. The solution was filtered to remove any insoluble material. The final concentration of Coomassie blue R250 was 0.25% (w/v). After electrophoresis, the apparatus was disassembled and the gel was immersed in the CBB solution. The gel was stained at room temperature overnight with gentle agitation. The Coomassie stain was subsequently removed by aspiration after staining. The gel was immersed in a destaining medium of methanol/acetic acid/water (5:1:4, v/v/v), in which it was destained with gentle agitation. The destaining step was repeated several times, removing the destaining medium each time by aspiration. The destaining was continued until the protein bands were seen clearly without any background staining of the gel.

Table S1. Fluorescent probes for thiol labeling.

Probes	Ex/nm	Em/nm	Analyte	LOD	Application	Response time	Reference
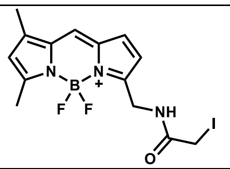	360	510	GSH	0.02 nM	HPLC	0.5 min	[1]
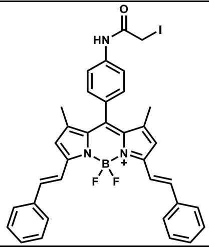	620	630	GSH	0.24 nM	HPLC	25 min	[2]
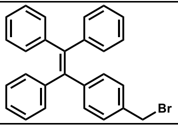	320	475	BSA	-	SDS-PAGE	3 h	[3]
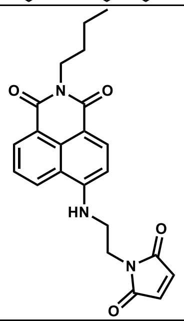	450	540	GSH	3.54 nM	Living cells, SDS-PAGE	3 min	[4]

biosensors

MDPI

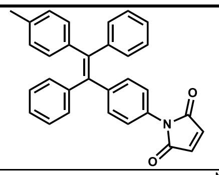	350	470	GSH	-	Living cells, SDS-PAGE	30 min	[5]
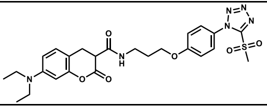	425	465	BSA	9.8 nM	Living cells	15 min	[6]
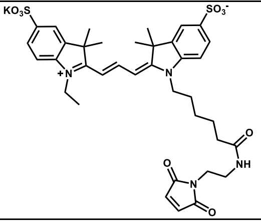	548	563	peptide	-	HPLC	overnight	[7]
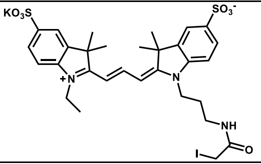	548	563	NAMPT-E263C	-	-	8h	[8]
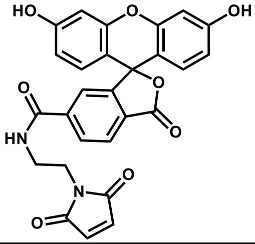	494	520	FB-E25FPheK-F6C protein	-	SDS-PAGE	12h	[9]
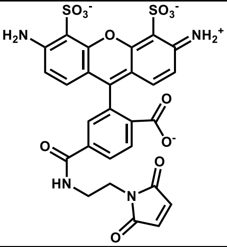	494	517	FB-E25FPheK protein	-	SDS-PAGE	12h	[9]
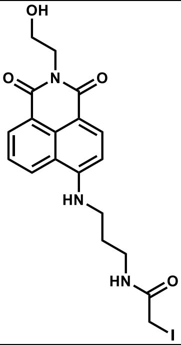	449	553	BSA	37.5 nM	SDS-PAGE HPLC	30 min	Our work

2. NMR and Mass Spectra

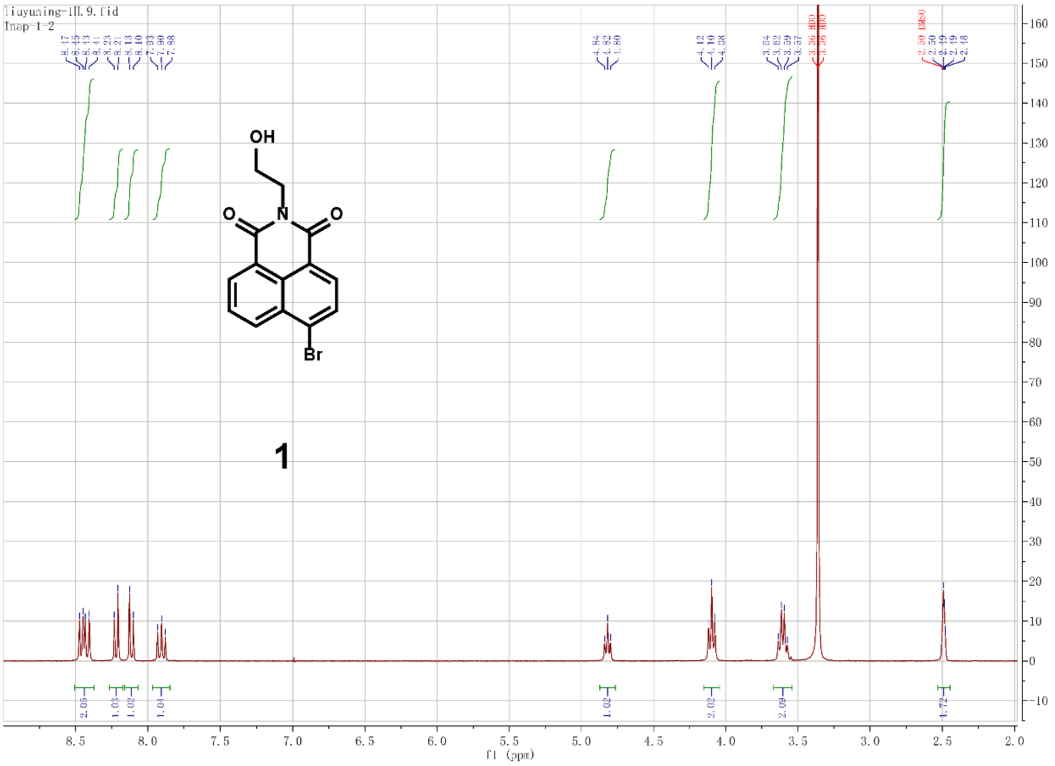

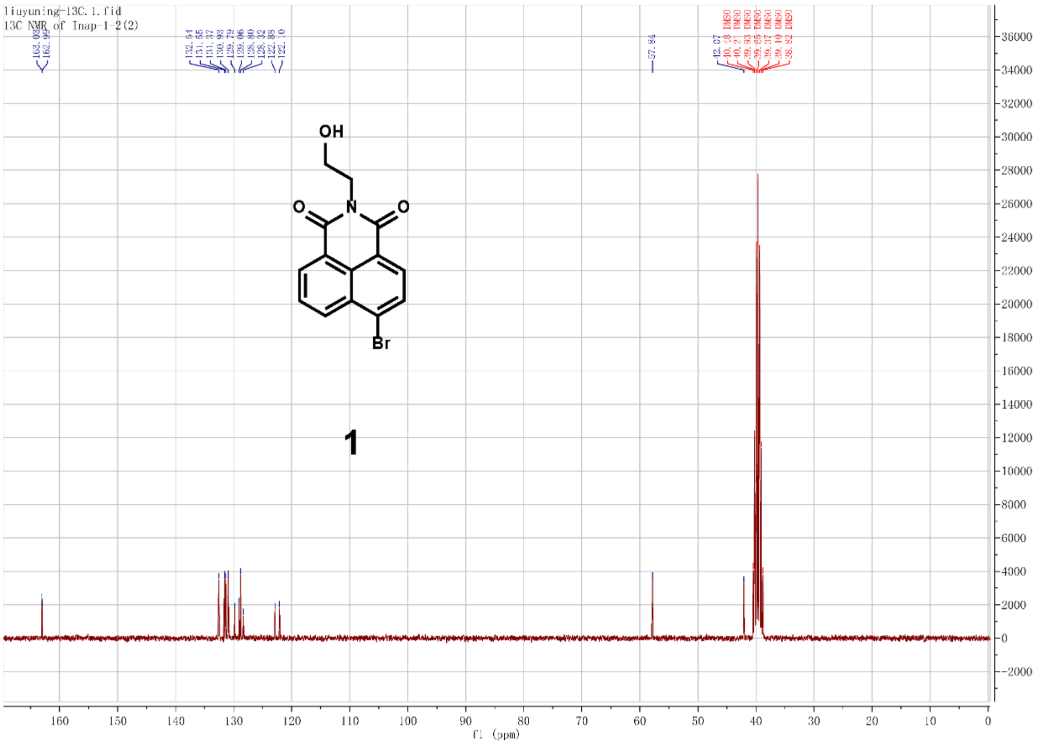

Figure S1. ^{1}H and ^{13}C NMR spectra of compound 1.

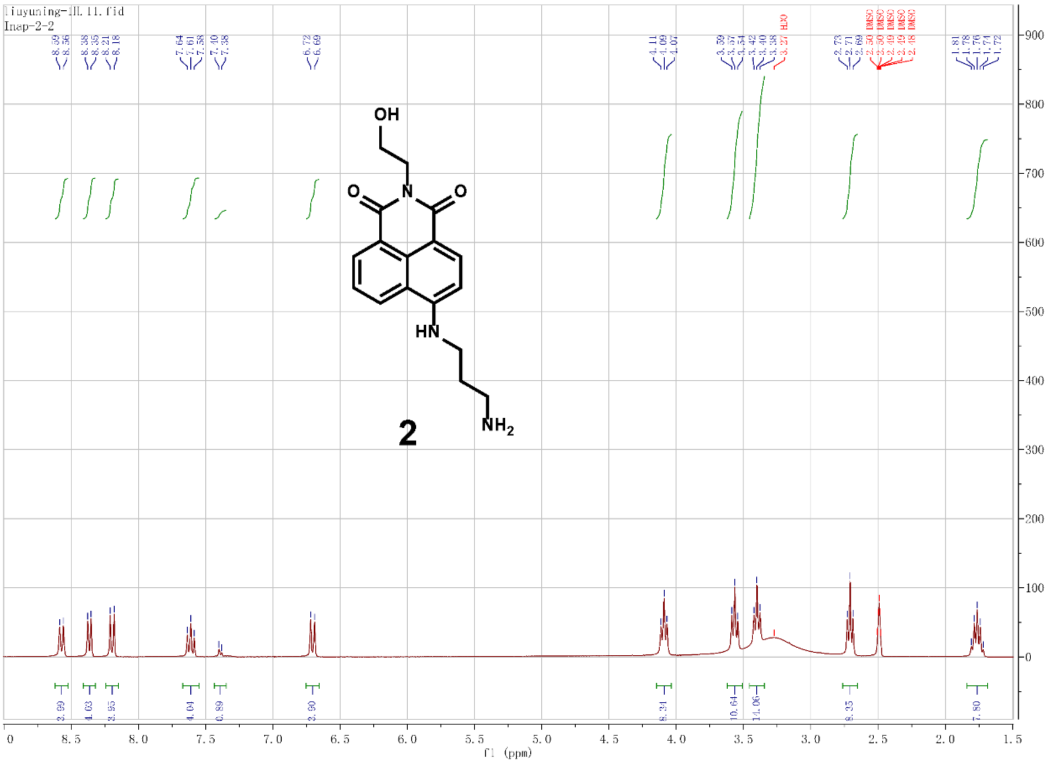

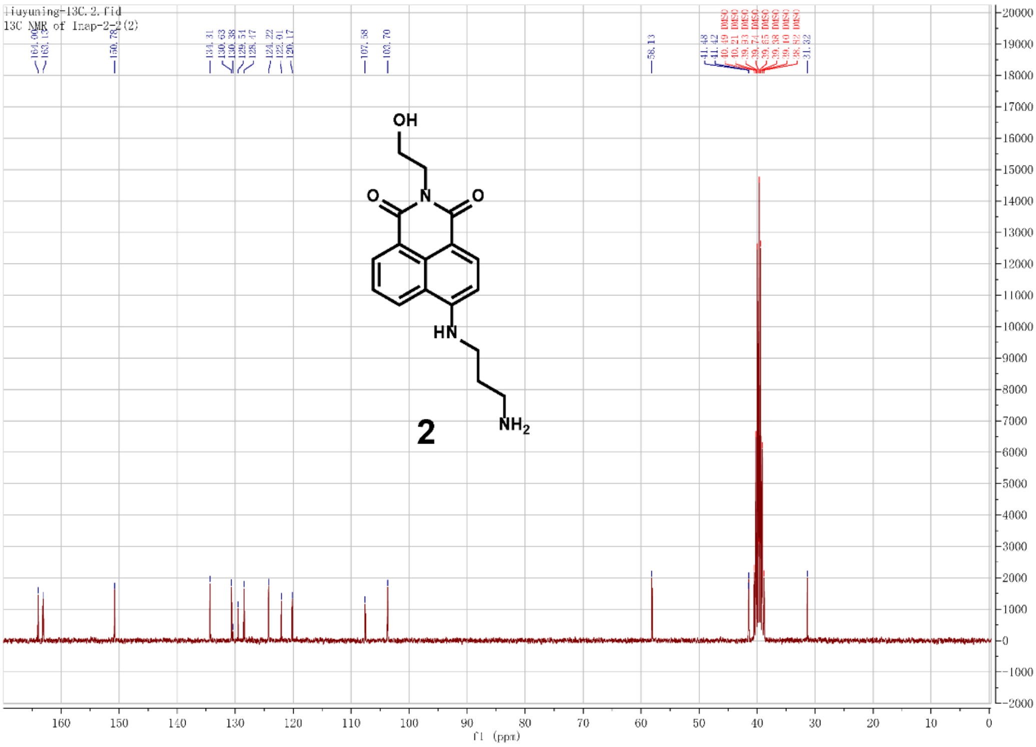

Figure S2. ^{1}H and ^{13}C NMR spectra of compound 2.

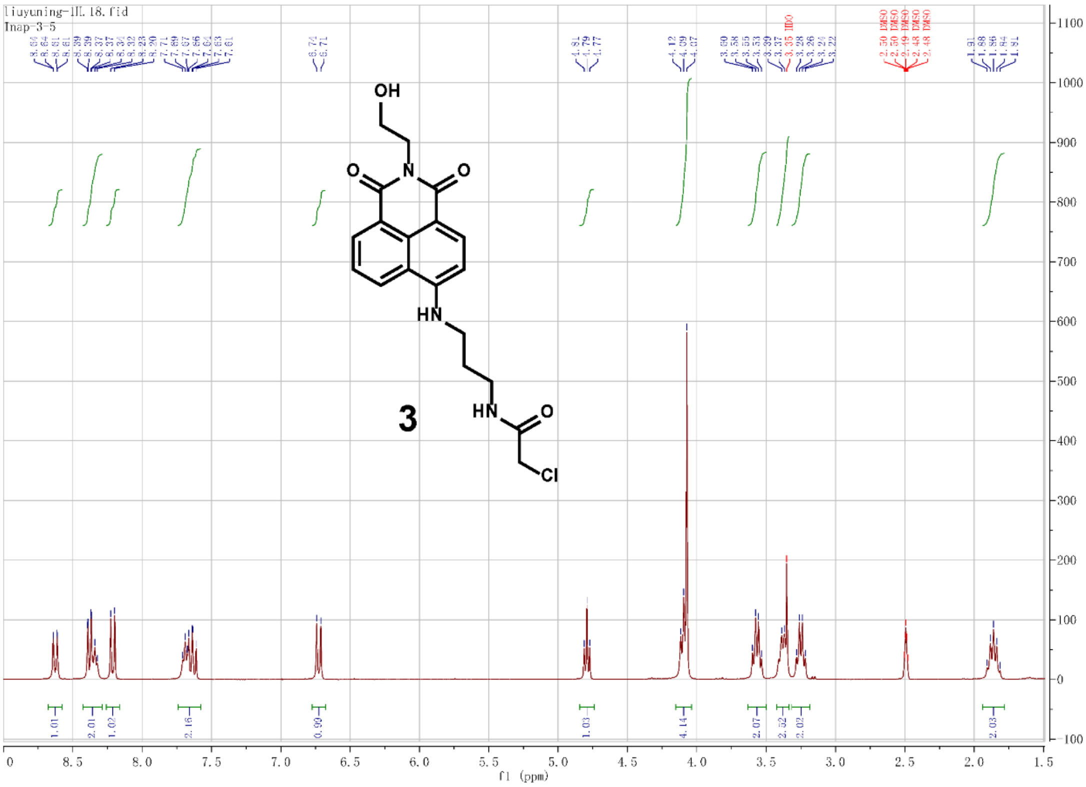

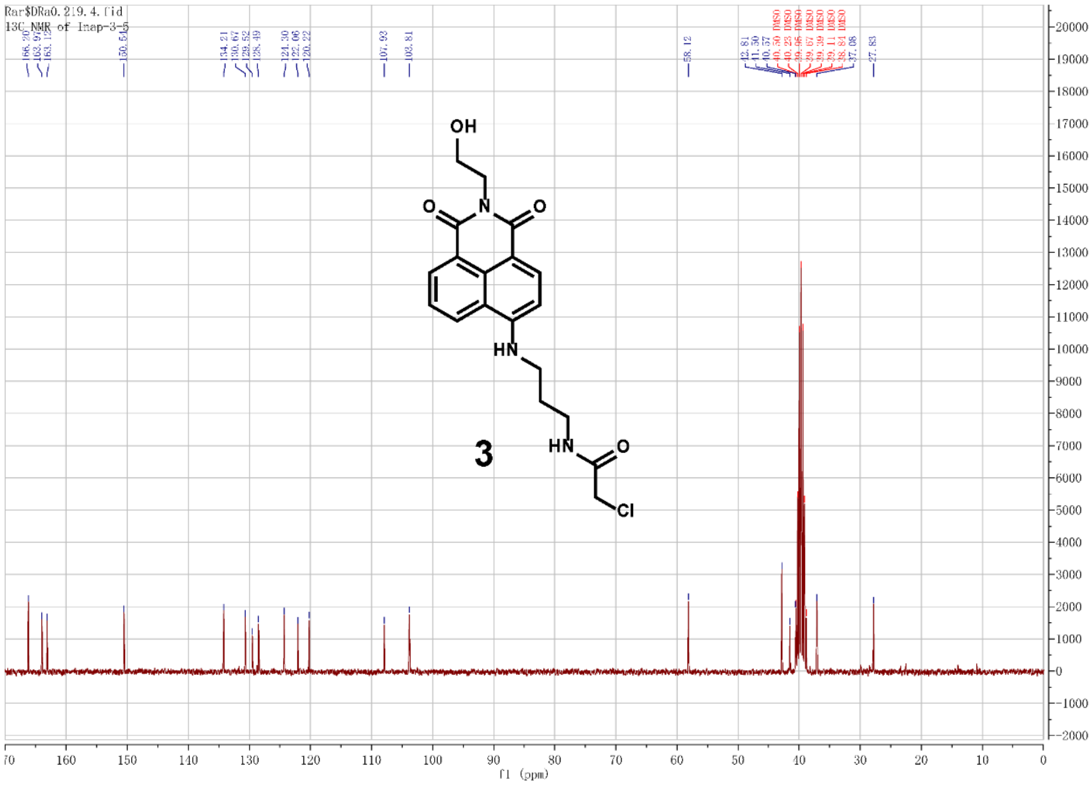

Figure S3. ^{1}H and ^{13}C NMR spectra of compound 3.

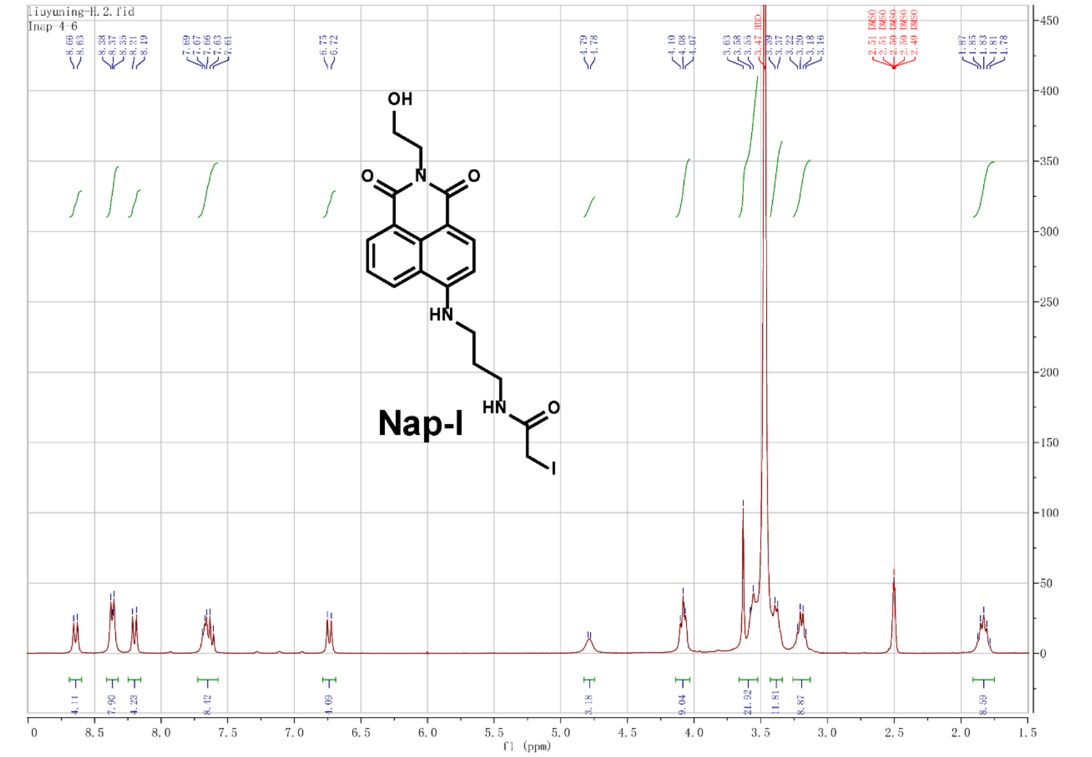
liuyuning-H.2.fid
Inap-4-6
Nap-I
OH
N
O
O
HN
HN
O
I
f1 (ppm)

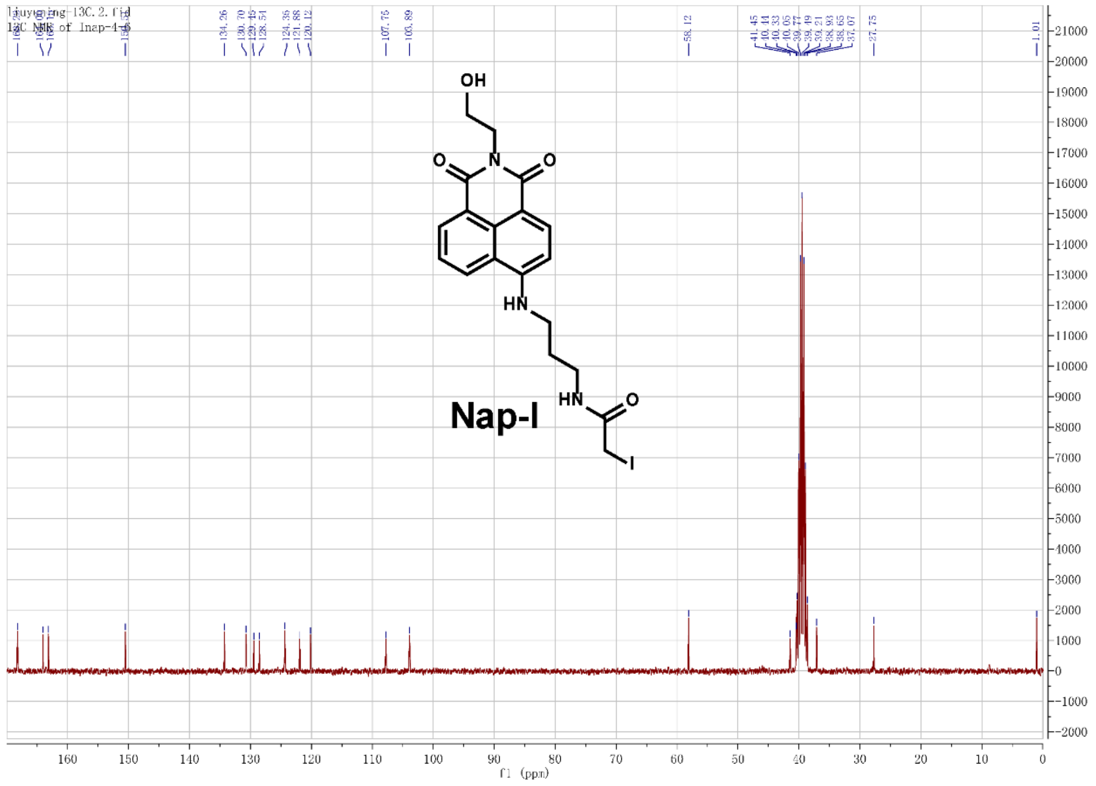
Nap-I
OH
N
O
O
HN
HN
O
I
f1 (ppm)

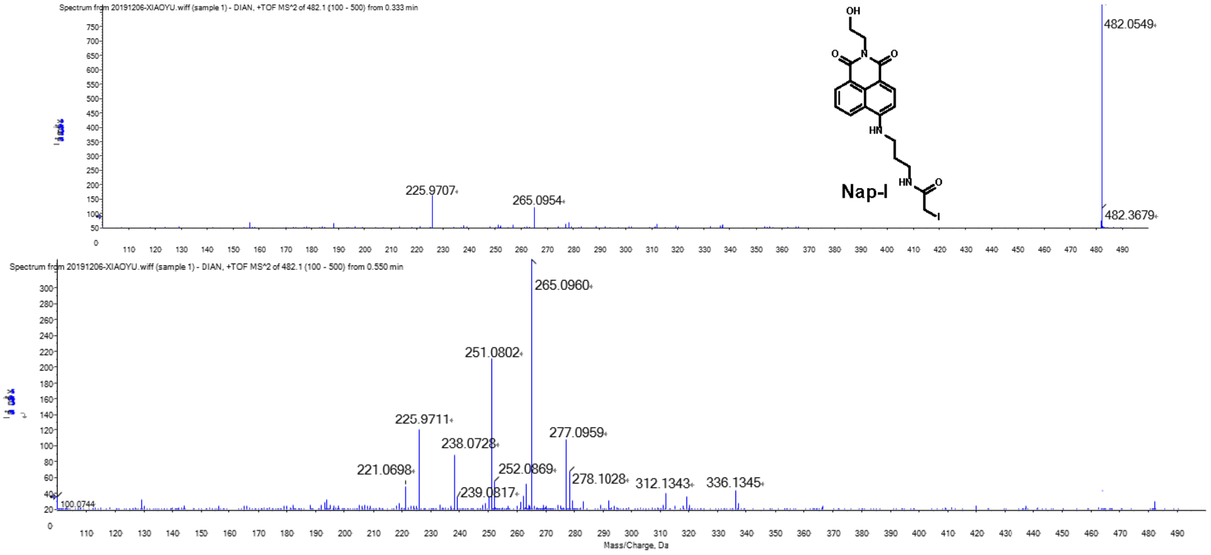

Figure S4. ^{1}H and ^{13}C NMR spectra and mass spectrum of Nap-I.

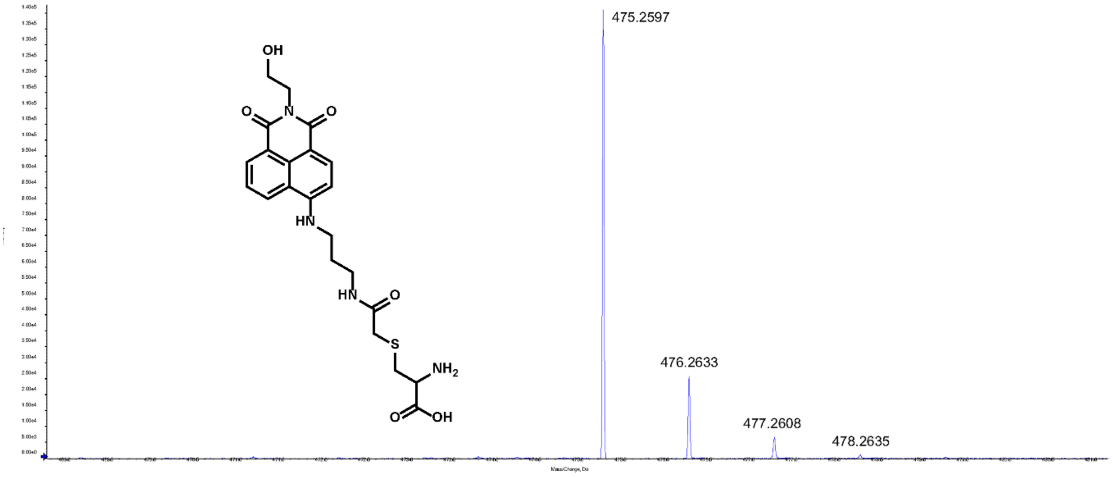

Figure S5. Mass spectrum of the alkylation product of Nap-I and Cys.

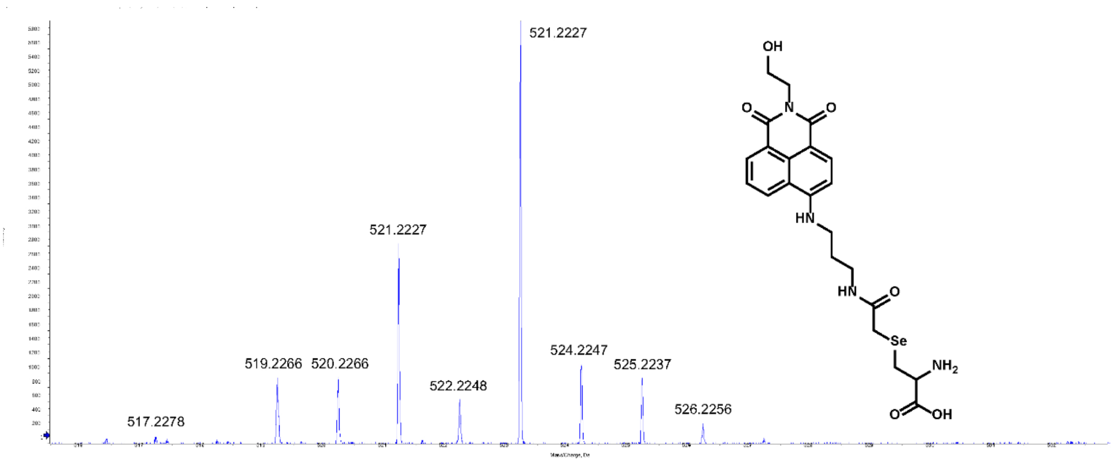

Figure S6. Mass spectrum of the alkylation product of Nap-I and Sec.

3. Fluorescence Spectra and SDS-PAGE Images

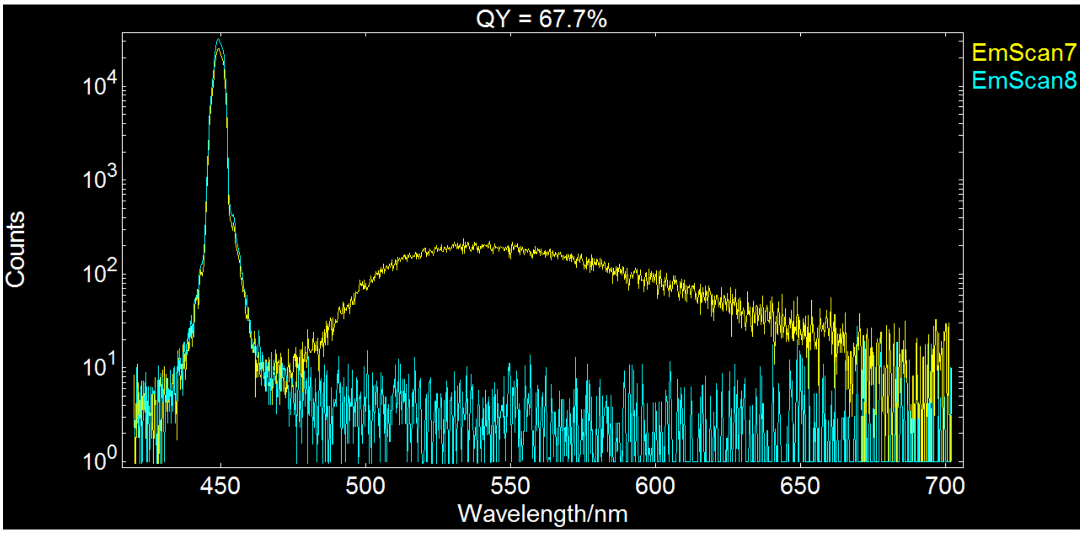

Figure S7. Quantum yield of Nap-I.

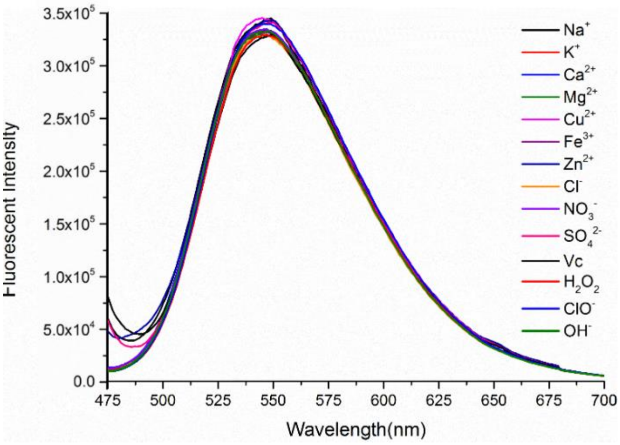

Figure S8. Interference experiments: fluorescence responses of 100 μM Nap-I in 100 mM Tris-HCl buffer with 1% DMSO as co-solvent (V_{Water}/V_{DMSO} = 99:1) in the presence of 10 mM of potentially interfering species, namely NaCl, KCl, $CaCl_2$, $MgCl_2$, $CuSO_4$, $FeCl_3$, $ZnCl_2$, HCl, HNO_3, H_2SO_4, vitamin C, H_2O_2, HClO, and NaOH.

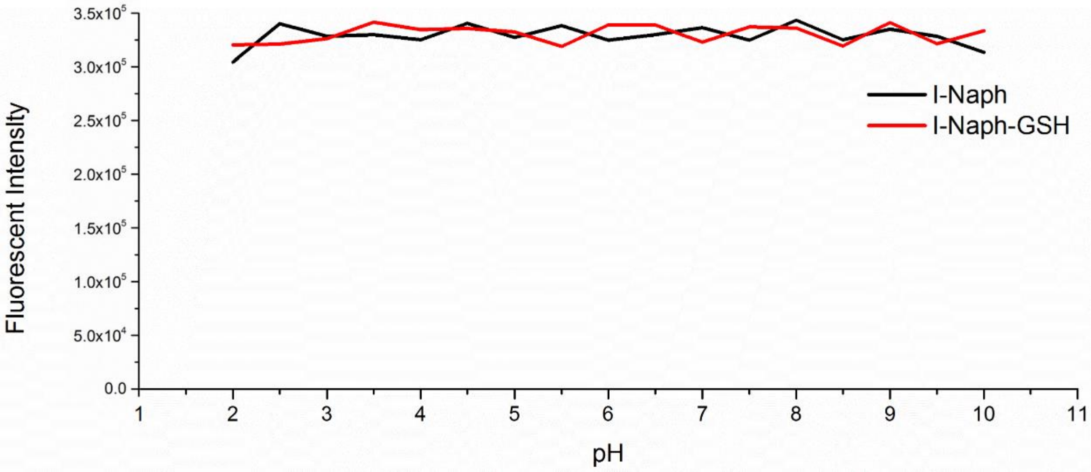

Figure S9. pH stabilities of 100 μM Nap-I and 100 μM Nap-GSH.

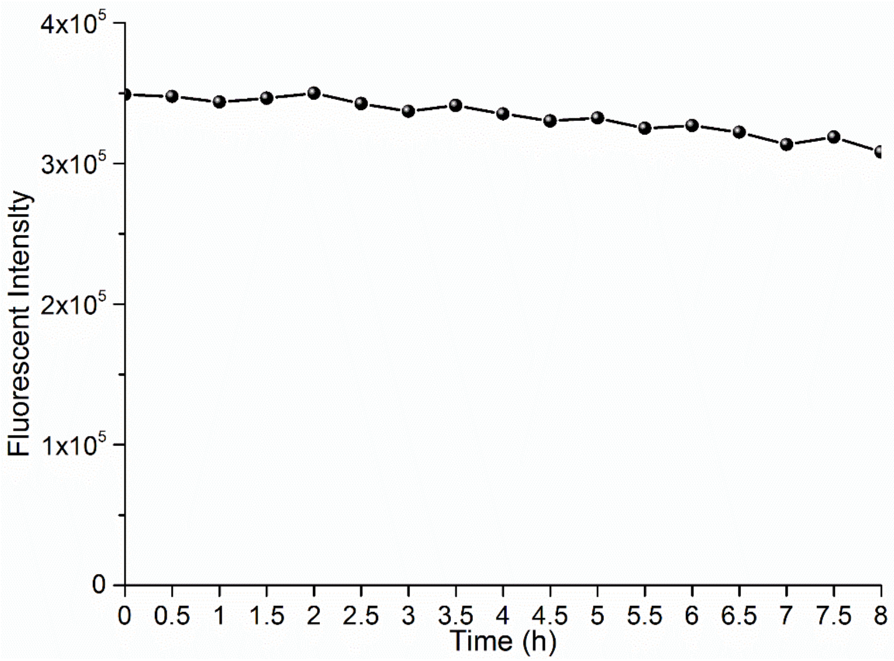

Figure S10. Photostability of 100 μM Nap-GSH.

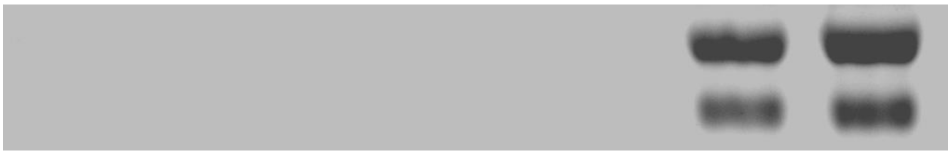

Figure S11. SDS-PAGE fluorescence image of Nap-I-labeled main proteins with different labeling pH (reaction in 30 min).

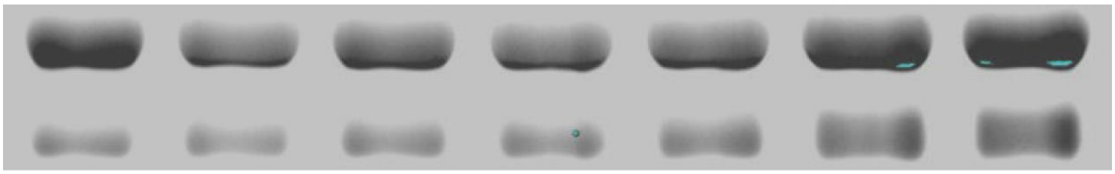

Figure S12. SDS-PAGE fluorescence image of Nap-I-labeled main proteins with different labeling pH (reaction in 1 h).

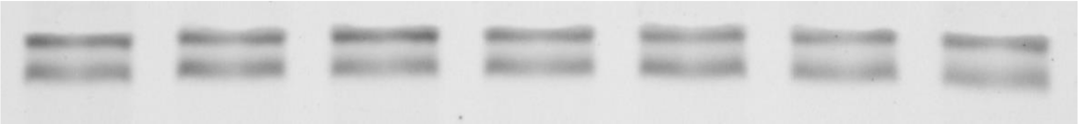

Figure S13. SDS-PAGE fluorescence image of Nap-I-labeled TXNRD from mouse liver reduced by NADPH with different labeling pH (reaction in 1 h).

References

1. Huang, K. J.; Han, C. H.; Han, C. Q.; Li, J.; Wu, Z. W.; Liu, Y. M., Determination of thiol compounds by solid-phase extraction using multi-walled carbon nanotubes as adsorbent coupled with high-performance liquid chromatography-fluorescence detection. *Microchim. Acta* **2011**, *174*, 421–427.
2. Zhang, L. Y.; Tu, F. Q.; Guo, X. F.; Wang, H.; Wang, P.; Zhang, H. S., A new BODIPY-based long-wavelength fluorescent probe for chromatographic analysis of low-molecular-weight thiols. *Anal. Bioanal. Chem.* 2014, *406*, 6723–6733.
3. Yu, Y.; Li, J.; Chen, S.; Hong, Y.; Ng, K. M.; Luo, K. Q.; Tang, B. Z., Thiol-reactive molecule with dual-emission-enhancement property for specific prestaining of cysteine containing proteins in SDS-PAGE. ACS Appl. *Mater. Interfaces* **2013**, *5*, 4613–4616.

4. Sun, J.; Zhang, L.; Zhang, X.; Hu, Y.; Ge, C.; Fang, J., An ultrafast turn-on thiol probe for protein labeling and bioimaging. *Analyst* **2016**, *141*, 2009–2015.
5. Chen, M. Z.; Moily, N. S.; Bridgford, J. L.; Wood, R. J.; Radwan, M.; Smith, T. A.; Song, Z.; Tang, B. Z.; Tilley, L.; Xu, X.; Reid, G. E.; Pouladi, M. A.; Hong, Y.; Hatters, D. M., A thiol probe for measuring unfolded protein load and proteostasis in cells. *Nat. Commun.* **2017**, *8*, 474.
6. Li, X.; Feng, Q.; Qu, L.; Zhao, T.; Li, X.; Bai, T.; Sun, S.; Wu, S.; Zhang, Y.; Li, J., A water-soluble and incubate-free fluorescent environment-sensitive probe for ultrafast visualization of protein thiols within living cells. *Anal. Chim. Acta* **2020**, *1126*, 72–81.
7. Allen, C. D.; Chen, M. Y.; Trick, A. Y.; Le, D. T.; Ferguson, A. L.; Link, A. J., Thermal Unthreading of the Lasso Peptides Astexin-2 and Astexin-3. *ACS Chem. Biol.* **2016**, *11*, 3043–3051.
8. Yang, Y.; Tang, C.; Gu, X., Synthesis of Near-Infrared Fluorescence Probe Reagents for Site-Specific Labeling of Proteins. *Chem. Bull.* **2016**, *79*, 856–875.
9. Yu, C.; Tang, J.; Loredo, A.; Chen, Y.; Jung, S. Y.; Jain, A.; Gordon, A.; Xiao, H., Proximity-Induced Site-Specific Antibody Conjugation. Bioconjug. *Chem.* **2018**, *29*, 3522–3526.
